# Proof of concept: used malaria rapid diagnostic tests applied for parallel sequencing for surveillance of molecular markers of anti-malarial resistance in Bissau, Guinea-Bissau during 2014–2017

**DOI:** 10.1186/s12936-019-2894-8

**Published:** 2019-07-26

**Authors:** Sidsel Nag, Johan Ursing, Amabelia Rodrigues, Marina Crespo, Camilla Krogsgaard, Ole Lund, Frank M. Aarestrup, Michael Alifrangis, PouL-Erik Kofoed

**Affiliations:** 1 Centre for Medical Parasitology, Department of Immunology and Microbiology, University of Copenhagen, Copenhagen, Denmark; 2 Department of Infectious Diseases, Copenhagen University Hospital, Copenhagen, Denmark; 3 Department of Clinical Sciences, Karolinska Institutet, Danderyds Hospital, Stockholm, Sweden; 4 Department of Infectious Diseases, Danderyds Hospital, Danderyd, Sweden; 5 Bandim Health Project, Bissau, Guinea-Bissau; 6 DTU Bioinformatics, Technical University of Denmark, Lyngby, Denmark; 7 Division for Epidemiology and Microbial Genomics, National Food Institute, Technical University of Denmark, Kongens Lyngby, Denmark; 8 Department of Paediatrics, Kolding Hospital, University of Southern Denmark, Kolding, Denmark

**Keywords:** *Plasmodium falciparum*, Guinea-Bissau, Molecular markers of antimalarial resistance, Rapid diagnostic tests, Next-generation sequencing, Amplicon sequencing, *pfcrt*, *pfmdr1*, *pfdhfr*, *pfdhps*, *pfk13*

## Abstract

**Background:**

Large-scale surveillance of molecular markers of anti-malarial drug resistance is an attractive method of resistance monitoring, to complement therapeutic efficacy studies in settings where the latter are logistically challenging.

**Methods:**

Between 2014 and 2017, this study sampled malaria rapid diagnostic tests (RDTs), used in routine clinical care, from two health centres in Bissau, Guinea-Bissau. In order to obtain epidemiological insights, RDTs were collected together with patient data on age and sex. A subset of positive RDTs from one of the two sites (n = 2184) were tested for *Plasmodium* DNA content. Those testing positive for *Plasmodium* DNA by PCR (n = 1390) were used for library preparation, custom designed dual indexing and next generation Miseq targeted sequencing of *Plasmodium falciparum* genes *pfcrt*, *pfmdr1*, *pfdhfr*, *pfdhps* and *pfk13*.

**Results:**

The study found a high frequency of the *pfmdr1* codon 86N at 88–97%, a significant decrease of the *pfcrt* wildtype CVMNK haplotype and elevated levels of the *pfdhfr/pfdhps* quadruple mutant ranging from 33 to 51% between 2014 and 2017. No polymorphisms indicating artemisinin tolerance were discovered. The demographic data indicate a large proportion of young adults (66%, interquartile range 11–28 years) presenting with *P. falciparum* infections. While a total of 5532 gene fragments were successfully analysed on a single Illumina Miseq flow cell, PCR-positivity from the library preparation varied considerably from 13 to 87% for different amplicons. Furthermore, pre-screening of samples for *Plasmodium* DNA content proved necessary prior to library preparation.

**Conclusions:**

This study serves as a proof of concept for using leftover clinical material (used RDTs) for large-scale molecular surveillance, encompassing the inherent complications regarding to methodology and analysis when doing so. Factors such as RDT storage prior to DNA extraction and parasitaemia of the infection are likely to have an effect on whether or not parasite DNA can be successfully analysed, and are considered part of the reason the data yield is suboptimal. However, given the necessity of molecular surveillance of anti-malarial resistance in settings where poor infrastructure, poor economy, lack of educated staff and even surges of political instability remain major obstacles to performing clinical studies, obtaining the necessary data from used RDTs, despite suboptimal output, becomes a feasible, affordable and hence a justifiable method.

**Electronic supplementary material:**

The online version of this article (10.1186/s12936-019-2894-8) contains supplementary material, which is available to authorized users.

## Background

In anticipation of novel emergence or geographic spread of especially artemisinin-resistant *Plasmodium falciparum* parasites [1–3], countries with malaria transmission are recommended by the World Health Organization (WHO), to test the efficacy of their recommended artemisinin-based combination therapy (ACT) every 2–3 years [4]. Therapeutic efficacy studies are often not feasible due to economic and practical constraints in many settings in sub-Saharan Africa (SSA). It has therefore been suggested that molecular surveillance of genetic polymorphisms associated with anti-malarial resistance [4–9] could complement therapeutic efficacy studies because these can provide early warning signs of decreasing anti-malarial efficacy [10]. Molecular surveillance only requires sampling of *P. falciparum* infected blood, which in turn can be acquired from finger prick samples. Malaria rapid diagnostic tests (RDTs), which are routinely used in clinical care in SSA, represent a massive body of finger prick blood samples, readily acquirable across SSA. Their use for molecular analyses is well described [11–13], but large-scale analyses using reliable, high throughput methodologies remains to be seen. Meanwhile, high throughput molecular analysis has become reliable and affordable with novel protocols based on next generation sequencing (NGS) technology [14–16]. If large-scale, high throughput molecular analysis of anti-malarial resistance markers is feasible on used RDTs, large-scale molecular surveillance of anti-malarial resistance can become reality.

The current study was carried out in the capital of Guinea-Bissau. Malaria epidemiology in Guinea-Bissau has changed during the past decades and is now highly seasonal with epidemics occurring from September, peaking in November and lasting through January [17, 18]. Children aged < 5 years no longer account for the majority of malaria cases as the median age is gradually increasing [18]. Since 2008, the first-line treatment for malaria has been artemether–lumefantrine (AL) [19], which was shown to be effective in the Bissau area [20]. Quinine (QN) is the second- and third-line treatment for malaria [19] and is, therefore, also used occasionally. Intermittent preventive treatment in pregnancy (IPTp), with sulfadoxine-pyrimethamine (SP), is implemented [19]. Lastly, during the study period dyhydroartemisinin–piperaquine (DP) was is in clinical trial being compared to AL for treatment of children.

The primary objective of this study was to evaluate whether used RDTs, collected after routine use in clinical care, can serve as source of parasite DNA for large-scale NGS-based molecular surveillance of anti-malarial resistance. Based on the hypothesis that parasite DNA can be extracted from used RDTs, a modified version of a previously published NGS-based amplicon sequencing methodology was used [15]. The molecular approach investigated to what extent SNPs and genes associated with tolerance/resistance towards the majority of currently available anti-malarial treatments could be analysed in this high-throughput manner, applying massive parallel sequencing and a custom-made sample-indexing approach. A secondary objective was to provide temporal molecular marker data from the setting in Bissau, Guinea-Bissau, based on the successful application of used RDTs for molecular surveillance. Samples were obtained from May 2014 until April 2017 together with minimal patient information in order to provide a basic description of the demographic trends amongst malaria patients versus non-malaria patients in the study area.

## Methods

### RDT sampling

Positive and negative RDTs were collected from patients of all ages whom health workers suspected might be infected with *P. falciparum* (typically associated with presence of a fever within the last 24 h), presenting at the Bandim or Belem health centres from May 2014 until April 2017. Patient age, sex and date of collection were written on the RDT and on a clinical records form. All information was put into a folder on a daily basis, and subsequently entered into an electronic database. RDTs were collected in a storage box containing silica gel, which was kept dark at room temperature (20–35 °C), and stored between 3 and 9 months before shipment to Denmark, where they were stored between 0 and 9 months at room temperature before DNA extraction was performed.

### Study size

**Fig. 1:**
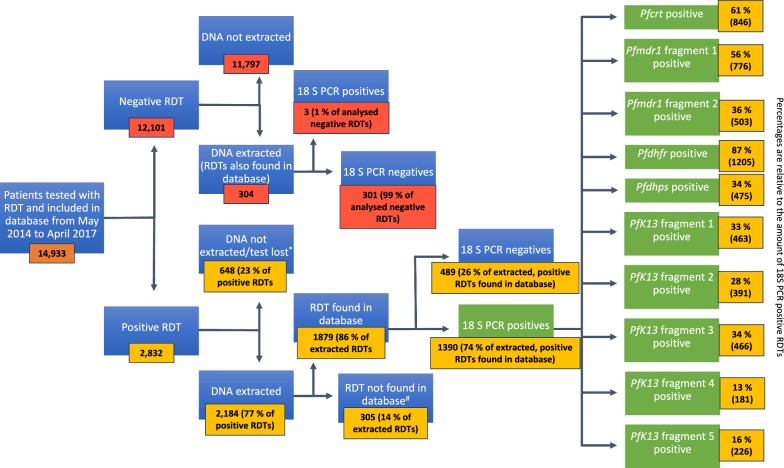
Sample screening and processing. Samples were collected from all patients tested with an RDT, and subsets of positive and negative samples were used for DNA extraction and subsequent analyses. *Almost all samples from Belem were lost due to faulty extraction procedures, and other samples logged in the database were never identified amongst the RDTs received. ^#^A number of positive RDTs received in Denmark, were not found in the database (no RDT with corresponding number or information had been logged). DNA extraction was performed before cross-referencing samples with the electronic database because the electronic database was not ready when samples were received, and postponing DNA-extraction was avoided

The study size varies throughout the study, with decreasing study size as the study progresses, because samples fail to meet the developing study criteria. Please view Fig. 1 for a visualization of the following: The initial study size was based on RDTs collected at the study sites, which were included in the database received in Denmark. RDTs were collected based on the criteria listed above (RDT sampling). The included samples were then categorized according to whether samples were positive or negative at the time of diagnosis (and hence listed as such in the database). Subsequently, the study size is massively reduced, as DNA is only extracted from a small subset of the negative RDTs. Furthermore, although DNA was intended extracted from all of the positive RDTs, as subset was lost due to faulty extraction. The cause for the faulty extraction was never identified, however no DNA was acquired from the tests extracted during a specific batch. Furthermore, the identification number, as well as patient data visible on the extracted RDTs was double checked with that from the database, at which point, samples not identified in both were discarded. At this point, the study size for estimating successful DNA extraction from positive RDTs/false-negative RDTs was derived. The study size for the molecular analysis was based on the RDTs from which DNA was successfully extracted.

### DNA extraction

DNA was extracted by the chelex method, as described previously [21], in a 96-well format with no samples in lane 12 and 4 blanks dispersed between lanes 1 and 11.

### PCR-corrected RDT positivity and negativity

A PCR amplifying the multicopy ribosomal 18S subunit of all *Plasmodium* species was performed on all positive RDTs received in Denmark, as well as on 304 negative RDTs from the high transmission seasons of 2014 and 2015. The PCR protocol has been described previously [22].

### Genetic analysis

**Fig. 2:**
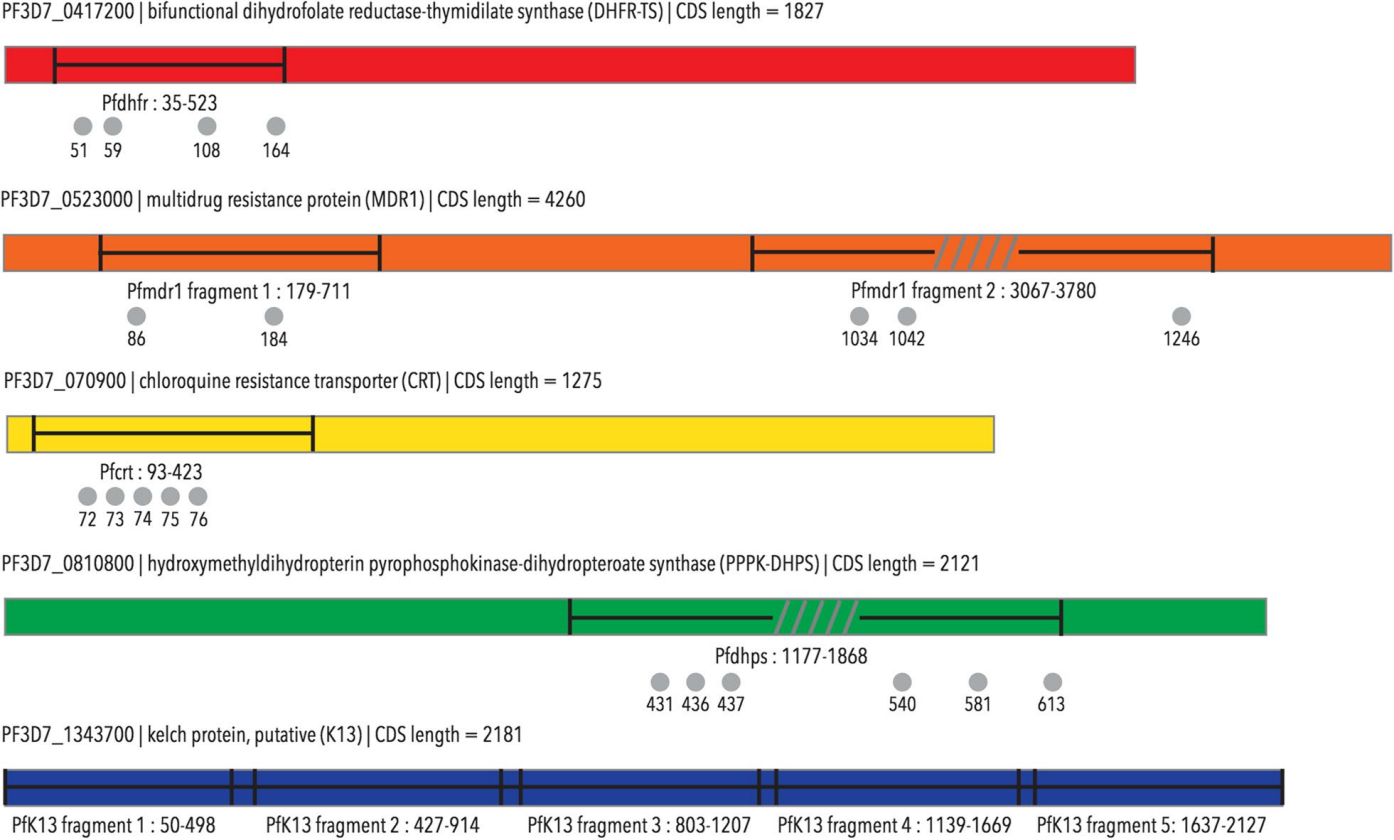
Amplicons incorporated in the sequencing library preparation. Fragments from *pfcrt*, *pfmdr1*, *pfdhfr*, *pfdhps* and *pfk13* were incorporated in the sequencing library. Grey circles indicate SNP positions of interest, and the numbering corresponds to codons. Grey lines indicate areas of amplicons that are not sequenced

Molecular markers of anti-malarial resistance were assessed by NGS-based amplicon sequencing, using a modified version of a previously published protocol [15]. In brief, amplicons (amplified gene-fragments) from the infecting parasites, depicted in Fig. 2, were produced by PCR and prepared for massively parallel sequencing on the Illumina Miseq sequencer through a previously published PCR-based library-preparation method [15] (based on Illumina’s own protocol for 16S metagenomics sequencing [23]). Primers synthesized for the previously published study, as well as some newly synthesized primers were used (see section regarding “Library preparation”). All amplicons pertaining to the same infection were barcoded with the same unique set of custom-made indices in the 5′ and 3′ ends. All barcoded amplicons were pooled prior to sequencing and sequenced in parallel. De-multiplexing of sequence data was performed based on all of the unique index-combinations given to the samples during library preparation. The original multiplex, non-nested amplification of gene-fragments were modified to simplex, nested amplification of the same or slightly modified gene-fragments (Fig. 2) due to the minute amount of DNA contained in RDT extracts [15]. As all PCRs were performed in simplex, certain fragments were redesigned to accommodate all of the genetic positions of interest within a single fragment (to reduce the number of PCRs), instead of two fragments (regarding *pfdhps* and 3′-*pfmdr1*). In these cases, certain areas of the fragments are not sequenced, as the paired-end 300 bp sequencing is not long enough to sequence the entire fragment. All primers are listed in Additional file 1: Table S1.

### Controls and duplicates

The majority of samples were run once, with 10% of samples run as duplicates. Control samples used in the study consisted of DNA from well characterized parasite-lines (viable in culture), namely 3D7, FCR3, DD2, K1, 7G8, MRA-1238 and MRA-1239 [9], the latter two of which are *pfk13* controls. Other controls consisted of patient samples from earlier studies, where the haplotypes within specific genes are known, namely AA (*pfdhps* 436A + 437A), AG (*pfdhps* 436A + 437G), 540E (*pfdhps* 540E) and 164L (*pfdhfr* 164L). An entire overview of control sample haplotypes is listed in Additional file 1: Table S2.

### Library preparation

A series of optimization attempts were performed to increase the PCR-positivity rate for the amplicons incorporated in the library. These attempts included (1) going from multiplex to simplex PCRs, (2) gradients on annealing temperatures (3) two-step annealing with an initial annealing of 40–44 °C for 5 cycles followed by annealing of 55–60 °C for 20–35 cycles, (4) increased cycle number during annealing (5) increased annealing and extension times and finally (6) altered primer sites. The optimizations were performed on all of the individual amplicons, with little to no effect. In conclusion,

PCRs were performed as described previously [15], with the following alterations: all fragments were amplified individually, and as nested PCRs, requiring new primer design for some of the primers. The outer and the nested PCR programs were identical to the previously published “gene-specific PCR”, except that they consisted of 40 cycles each. The nested PCR was performed with primers containing the overhangs, as was previously the case for the “gene-specific PCR”. The nested PCR products were pooled according to sample of origin, prior to index PCR, as described previously. The index PCR was run according to the original protocol [15]. All primers and corresponding fragments are listed in Additional file 1: Table S1, and depicted in Fig. 2. Fragments included in the study cover *pfcrt* codon (c) c. 31–138, *pfmdr1* c. 60-237 and c. 1022-1260 (where c. 1123-1160 are not sequenced), *pfdhfr* c. 12-174, *pfdhps* c. 392-622 (where c. 492-522 are not sequenced) as well as *pfK13* c. 17-709.

Amplicon purification, dilution, pooling and sequencing were all performed as previously described, at the DTU Multi Assay Core (DMAC), Technical University of Denmark [15].

### Quality trimming and base calling

Data was demultiplexed by the sequencer, using a custom-sample sheet (the amount of samples analysed in this study cannot be demultiplexed by BaseSpace, the run is visualized as faulty). Quality trimming of raw sequencing reads was performed using *cutadapt* [24], removing the 5′-primer sequence of every read, and setting a quality cut-off of 20. Data analysis was performed with *assimpler* [25], as described previously [15]. Briefly: *assimpler* is a python program which compares reads to a custom database. The program was set to call bases with a minimum z-score of 1.96.

### Mixed infections

Infections were defined as mixed if more than one base was called for a given position in a given sample, and was supported by at least 25% of the calls for that position for the sample in question. A minimum of 25% of calls was set as threshold, because a certain amount of calls is required to support the given base call. With the amount of samples simultaneously sequences on the flow cell, there will be variation in the amount of calls per position per sample.

### Statistics

Pearson’s Chi square was used to assess whether there was a difference in the proportions of children and adults amongst RDT-positive patients as compared to the general population. Fisher’s exact test was used to assess whether there was a significant trend over time in the frequencies of the detected haplotypes. Mixed infections were counted in all groups for single SNP-prevalence and omitted for haplotypes.

### Bias

Two main elements represent theoretical bias in this study. However, they have either been discussed as bias in “Discussion” section or addressed in the methodology.

1. *Bias within the sample population*: All samples were acquired at a health care centre and samples are assumed to be derived from symptomatic patients. This introduces a demographic bias, as some demographic groups are less likely to experience symptoms (elderly people with high acquired immunity and adults in general with very low-parasitaemic infections).
2. *Bias within the gene sequences*: In order to successfully analyse the genetic sequence of the genes of interest to anti-malarial resistance, specific primers have been designed to amplify the genes of interest. First of all, if there is genetic variation within the primer sequence, the gene is either not amplified for the specific sample, or the genetic variation will not be identified if successfully amplified. The latter is due to the primer sequence being trimmed from the sequences analysed, in order to not introduce primer-bias.

## Results

### Evaluation of the applicability of RDTs as source of DNA for NGS-based molecular surveillance

#### PCR-corrected RDT positivity and negativity

In total, 14,933 RDTs were used to diagnose patients at the two health centres between May 2014 and April 2017, and collected. Out of these, 2832 RDTs were positive. A flow chart depicting the sample screening and selection process is shown in Fig. 1. The majority of positive samples collected at the Belem health centre were lost due to a faulty extraction procedure. In order to not introduce bias in into the results, it was decided to only go forward with samples from Bandim for PCR analysis. All positive RDTs collected at the Bandim health centre which were received in Denmark (n = 2184, 77% of the RDT positive samples) were subjected to DNA extraction. Samples that were successfully found logged in the RDT database (n = 1879, 86% of the DNA-extracted samples) were then checked for PCR-positivity of the ribosomal 18S *Plasmodium* subunit. The overall PCR-corrected positivity amongst these samples was 74% (n = 1390).

A total of 304 negative RDTs from the 2014 and 2015 transmission periods, collected at the Bandim health centre, were also tested for PCR-positivity. Only 1% of these (n = 3) were found to be PCR-positive.

#### PCR success-rate for single copy genes during library preparation

In total, 1390 PCR corrected *Plasmodium* 18S-positive samples were used in nested PCRs designed to amplify the various gene fragments analysed in this study. The success-rate of single-copy gene PCRs varied from 13 to 87% (Fig. 1). Specifically, the *pfdhfr* fragment was successfully amplified for 87% of 18S-positive RDTs, while *pfcrt* and *pfmdr1* fragment 1 were amplified for 61% and 56%. The *pfmdr1* fragment 2, *pfdhps* as well as *pfk13* fragments 1–3 were all amplified for 28–36%, and lastly, *pfk13* fragments 4 and 5 were amplified for 13% and 16%, respectively. In total, 5532 gene fragments were successfully sequenced.

### Molecular markers of anti-malarial resistance

The observed frequencies of specific haplotypes are listed in Table 1 (mixed infections were omitted from haplotype analyses), while single SNP frequencies are listed in Table 2.

**Table 1:** Frequencies of haplotypes found during the transmission periods 2014–2016 (May 2014–April 2017), p-values are from Fisher’s exact test for trend over time

Frequencies of haplotypes					
Gene	Haplotype	2014	2015	2016	p-value (trend)
18S success		76% (n = 228)	80% (n = 676)	66% (n = 486)	
*pfcrt* c. 72-76	CVMNK (wildtype)	67% (45/67)	60% (187/311)	45% (99/219)	0.006
	CVIET (mutant)	33% (22/67)	40% (124/311)	55% (120/219)	0.006
*pfmdr1* c. 86 + 184	NF	57% (43/75)	73% (212/290)	62% (116/187)	0.052
	NY	29% (22/75)	23% (68/290)	29% (55/187)	0.052
	YF	11% (8/75)	3% (9/290)	9% (16/187)	0.084
	YY	3% (2/75)	0% (1/290)	0% (0/187)	0.109
*pfdhfr* c. 51 + 59 + 108	NCS (wildtype)	17% (14/82)	4% (18/407)	4% (17/443)	0.001
	IRN (triple mutant)	73% (60/82)	92% (373/407)	85% (376/443)	0.001
*pfdhps* c. 436 + 437 + 540 + 581 + 613	AAKAA (wildtype)	25% (17/68)	25% (23/93)	23% (39/170)	0.956
	AGKAA (mutant)	15% (10/68)	6% (6/93)	11% (19/170)	0.439
	SAKAA (wildtype)	26% (18/68)	29% (27/93)	21% (36/170)	0.083
	SGKAA (mutant)	34% (23/68)	38% (35/93)	43% (73/170)	0.448
*pfdhfr* c. 51 + 59 + 108 + *pfdhps* c. 437	IRN + G (quadruple mutant)	33% (16/49)	49% (97/199)	51% (94/185)	0.072

Mixed infections were omitted from haplotype analysis

**Table 2:** SNP prevalence, mixed infections counted in both groups

Gene	Codon	Amino acid	2014		2015		2016	
			Percentage	Count	Percentage	Count	Percentage	Count
18S				228		676		486
*pfcrt*	74	M	74	64	67	267	48	120
		I	43	37	53	209	61	152
	75	N	74	64	67	267	48	120
		E	43	37	53	209	61	152
	76	K	74	64	68	271	48	120
		T	43	37	53	210	61	152
*pfmdr1*	86	N	88	70	97	351	92	202
		Y	14	11	4	13	13	29
	184	F	70	56	81	315	74	163
		Y	34	27	42	163	38	84
	1034	S	100	67	100	182	100	202
		C	0	0	0	0	0	0
	1042	N	100	67	100	182	100	202
		D	0	0	0	0	0	0
	1246	D	96	64	100	182	100	201
		Y	4	3	0	0	0	1
*pfdhfr*	51	N	28	24	9	40	16	80
		I	76	65	94	408	92	471
	59	C	29	25	11	47	19	96
		R	76	65	93	403	91	466
	108	S	20	17	7	35	7	40
		N	84	73	96	449	97	526
	164	I	100	86	100	432	100	513
		L	0	0	0	0	0	0
*pfdhps*	436	S	73	36	77	157	68	127
		A	31	15	30	62	36	67
	437	A	67	33	57	114	47	88
		G	33	16	53	107	58	109
	540	K	98	52	99	134	99	186
		E	2	1	1	2	1	2
	581	A	100	53	100	136	100	185
		G	0	0	0	0	0	0
	613	A	100	52	98	129	99	187
		S/T	0	0	2	2	1	1

#### SNPs in *pfcrt* and *pfmdr1*

**Fig. 3:**
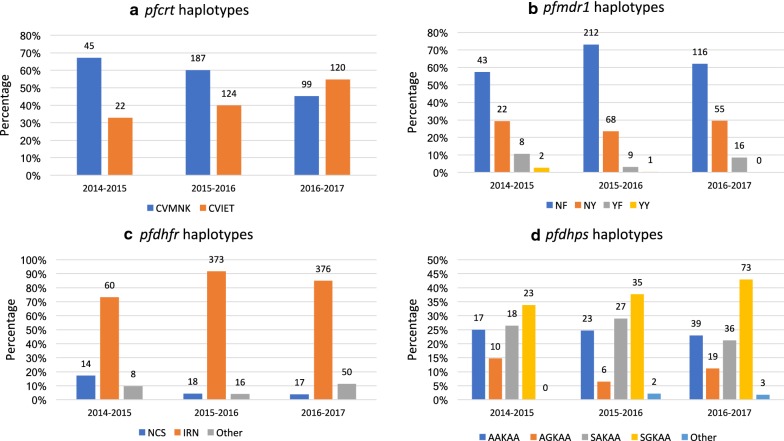
Molecular markers of anti-malarial resistance 2014–2017. **a** Frequency of *pfcrt* c. 72-76 haplotypes CVMNK and CVIET found each consecutive transmission season. **b** Frequency of *pfmdr1* c.86 + 184 haplotypes NF, NY, YF and YY found each consecutive year. **c** Frequency of *pfdhfr* c. 51 + 59 + 108 haplotypes IRN, NCS and “other” (consisting of NCN, ICN and NRN) found each consecutive year. The single mutant NCN represented 6/8 “other” *pfdhfr* haplotypes during the 2014 transmission season, while the two double mutants ICN and NRN combined accounted for 15/16 and 49/50 of “other” *pfdhfr* haplotypes found during the 2015 and 2016 transmission seasons, respectively. **d** Frequency of *pfdhps* c. 436 + 437 + 540 + 581 + 613 haplotypes AAKAA, AGKAA, SAKAA and SGKAA found each consecutive year

The *pfcrt* c. 72-76 CVMNK wild type was found to decrease significantly through the years of sampling; from 45/67 (67%) to 187/311 (60%) and to 99/219 (45%) samples in 2014, 2015 and 2016, respectively (p = 0.006, Fisher’s exact test) (Table 1, Fig. 3a). The *pfmdr1* c. 86 + 184 NF haplotype was found in 43/75 (57%), 212/290 (73%) and 116/187 (62%) samples, while the NY haplotype was found in 22/75 (29%), 68/290 (23%) and 55/187 (29%) samples (Table 1, Fig. 3b). SNP frequencies for *pfmdr1* c. 1034, 1042 and 1246 are listed in Table 2.

#### SNPs in pfdhfr and pfdhps

The *pfdhfr* c. 51 + 59 + 108 IRN triple mutant was found in 60/82 (73%), 373/407 (92%) and 376/443 (85%) samples in 2014, 2015 and 2016, respectively (p = 0.001, Table 1, Fig. 3c). The *pfdhps* c. 437G was found in 16/49 (33%), 105/199 (53%) and 109/185 (58%) samples during 2014, 2015 and 2016, respectively (mixed infections included). Accordingly, the quadruple *pfdhfr/pfdhps* mutant (*pfdhfr* IRN + *pfdhps* c. 437G) was found in 33%, 49% and 51% of samples. SNP frequencies for *pfdhps* c. 436, 540, 581 and 613 are listed in Table 2.

#### SNPs identified in pfk13

**Fig. 4:**
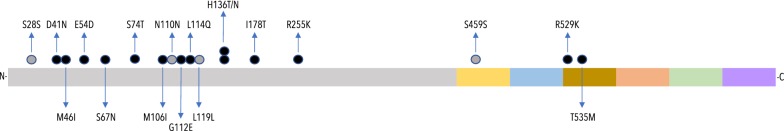
*pfk13* polymorphisms observed 2016–2017. Polymorphisms detected in *pfk13* during the transmission season from September 2016–January 2017. The grey bar indicates the N-terminal part of the translated K13 protein, while the coloured bars (yellow, blue, brown, peach, green and purple) indicate blades 1–6 in the propeller region. Grey circles indicate a synonymous SNP, while black circles indicate a non-synonymous SNP. Positions refer to amino-acid positions in the translated protein. The R529K and T535M mutations were each found only once

As PCR-positivity for *Pfk13* fragments was very low, only data from the latest of the transmission periods is presented. A total of 311 samples collected during the 2016 transmission period were partially or completely sequenced in *pfk13*, whereof 97 were successfully sequenced in the propeller region. In total, 18 SNPs were identified in *pfk13*, only 3 of which were situated in the propeller region, 2 of which are non-synonymous (R529K and T535M) (Fig. 4). In the N-terminal region, 15 SNPs were identified, 12 of which were non-synonymous (Fig. 4). None of the identified SNPs occurred in more than two samples.

### Demographic trends of RDT-positive versus RDT-negative patients

**Fig. 5:**
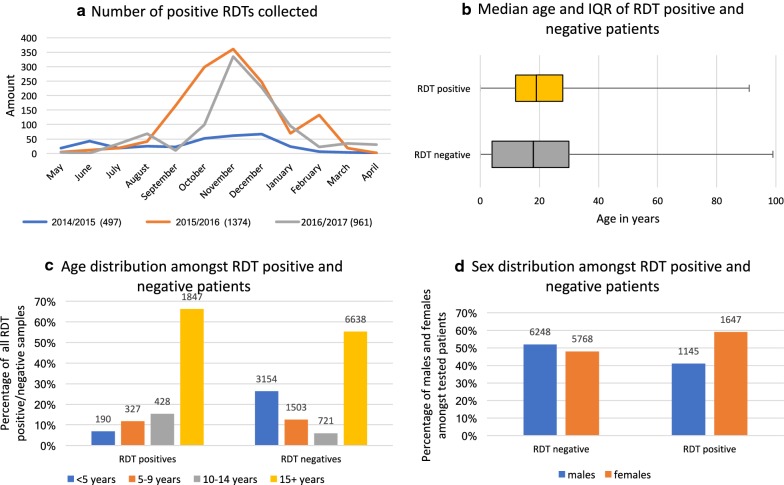
Description of RDT positive and negative patients included in the study. **a** Number of positive RDTs collected at the two health centres combined every month, for the three consecutive years of sampling, going from May to April. The malaria transmission season goes from September through January. **b** Median age and IQR of RDT positive and RDT negative patients included throughout the study. **c** Age distribution of RDT positive and RDT negative patients into groups consisting of < 5 years, 5–9 years, 10–14 years and ≥ 15 years. **d** Sex distribution of RDT positive and RDT negative patients throughout the study

Sampling was carried out for 36 months, starting May 2014. Transmission periods were therefore defined as periods of 12 months going from May 1 year up to and including April the following year, which includes the high transmission period September to January. In order to compare years and transmission periods, transmission periods have been named according to the year when transmission started. The number of positive RDTs collected during the 2014, 2015 and 2016 transmission periods were 497, 1374 and 961, respectively (Fig. 5a). The number of positive RDTs collected during the 2014 transmission period was substantially lower than the numbers collected in the two later transmission periods. Unexpected “dips” in the number of RDT positive patients were seen during January and September 2016 (Fig. 5a).

The median age of patients with a positive RDT was 19 years (interquartile range (IQR) 11–28) (Fig. 5b). When divided into age groups of < 5 years, 5–9, 10–14 and ≥ 15, the number of patients with positive RDTs were 190 (6%), 327 (12%), 428 (15%) [children less than 15 years of age combined = 945 (34%)] and 1847 (66%) (Fig. 5c, 40 samples did not have age stated). The sex distribution amongst RDT positive patients was 1145 males (52%) and 1647 females (48%) (Fig. 5d, 40 samples did not have sex stated).

The total number of negative RDTs collected was 12,101. The number of negative RDTs collected during the 2014, 2015 and 2016 transmission periods were 4001, 4362 and 3738 (RDT negative database only includes until February 19th 2017), respectively. The median age of patients with a negative RDT was 18 years (IQR = 4–30) (Fig. 5b). When divided into age groups of < 5 years, 5–9, 10–14 and ≥ 15, the number of patients with a negative RDT was 3154 (26%), 1503 (13%), 721 (6%) and 6638 (55%) (Fig. 5c, 85 samples did not have age stated). The sex distribution amongst RDT negative patients was 6248 males (52%) and 5768 females (48%) (Fig. 5d, 85 samples did not have gender stated).

In order to assess whether the proportion of adults was higher in the group of RDT positive patients than in the general population, proportions were compared to that of the general population of the country, estimated in 2015 as 41.7% children below the age of 15 vs 58.3% adults [26]. The proportion of adults within the group of RDT-positive patients was found to be significantly higher than that within the general population (Pearsons Chi square, p = 0.05), while the proportion of adults within the entire group of RDT-tested patients was not.

## Discussion

### Proof of concept

#### Applicability of the used RDTs for the molecular analyses

The study found that only 74% of the positive RDTs collected contain PCR-detectable *Plasmodium* DNA, which is required for molecular surveillance. This finding illustrates that the cost-efficiency of using RDTs for molecular surveillance is substantially affected by pre-screening the samples for the presence of *Plasmodium* DNA, as a notable percentage of samples is proven inapplicable for library preparation at this stage. Furthermore, the PCR-positivity of the single-copy genes involved in conferring resistance towards anti-malarial drugs varied tremendously from 13 to 87% after corrected 18S PCR-positivity. Let it be noted that the applied PCR protocols have been extensively optimized (see methods section) to increase positivity rates on RDT extracts. Studies using erythrocyte-enhanced samples [15] or dried venous blood spots (not erythrocyte-enhanced) on filter paper (Schmiegelow, Hansson, Nag and Alifrangis, unpublished) subjected to the same protocol resulted in PCR positivity of at least 90% for all fragments. Both the minute amount of parasite DNA available from an RDT, the DNA extraction protocol applied, as well as the state of the DNA in question (both at the time of extraction and at the time of running PCRs) may have contributed to the considerable variation in PCR positivity of single-copy genes. Unfortunately, the storage conditions and consequent degradation of DNA in used RDTs sampled at local health facilities, will be difficult to optimize in comparison to the protocols applied. However, a preliminary screening of the DNA extracts for parasitaemia/relative amount of parasite DNA available from the extracts, could allow for implementation of a minimum-value necessary for justification of library preparation from individual samples. While such an approach requires adding an extra qPCR step to the overall analysis, this qPCR step could actually replace the initial 18S screening step by providing information regarding to whether or not extracts contain parasite DNA, as well as indicate the relative amounts when doing so.

Overall, the analysis became more expensive per sample when using RDTs, than it would have been if samples had consisted of dried venous blood. This is due to several facts (1) library preparation consisted of simplex nested PCRs rather than multiplex non-nested PCRs, (2) primer concentrations applied were higher, (3) PCR programs were longer (machines ran for longer time) and (4) a considerable amount of samples were used for library preparation resulting in little to no data output. It was, however, still feasible to sequence 5532 gene fragments of approximately 500 bp, all with individual indices allowing trace-back to the sample of origin, on a single Miseq V3 flow cell with paired-end reads. With an approximate price for a Miseq V3 flow cell of USD 2300, this amounts to a sequencing cost per amplicon sequenced of USD 0.42. These costs do not include resources used during DNA extractions and PCR procedures, however they also do not reflect the resources saved from using spent RDTs rather than sampling venous blood spots. This study undoubtedly illustrates that many resources are used extracting DNA and running PCRs from samples that eventually will not result in any data output, illustrating that RDTs are not a perfect match for an NGS platform like the one described. However, due to the convenience and affordability in acquiring spent RDTs as well as the possibility of simultaneously and efficiently analysing a very large number of samples on a single flow cell through custom indexing, this study also illustrates that despite a massive loss in potential data output, combining RDTs with NGS still proves relatively affordable and efficient.

Other noteworthy limitations to using RDTs as illustrated by the current study, include the fact that routine sampling of RDTs is completely dependent on RDT availability. In our study, RDTs may have been out of stock during January and September 2016, where unexplained “dips” in malaria frequency are seen for periods of time, in which case inclusion numbers for these months would be biased. Furthermore, the nested PCR protocol which is required for the DNA extracted from RDTs, poses a much larger contamination risk during PCR procedures, than a non-nested PCR protocol [27, 28]. Finally, there are no sample backups when sampling RDTs, which may become a logistical and ethical concern, as samples can rather easily be completely lost until DNA is successfully extracted and backups created.

#### Molecular markers of anti-malarial resistance

The high prevalence of *pfmdr1* 86N in the current study, resembles previously published data for the same study area in 2010–2012 (approximately 80%) [29], indicating a relatively stable prevalence. The data corresponds well with the use of AL and the AL-derived selection of the *pfmdr1* c. 86N [30–32]. Importantly however, a recently performed efficacy study indicates that the efficacy of AL is still 94–95% [20], indicating that the prevalence of the *pfmdr1* c. 86N at levels between 88 and 97% is not affecting AL treatment efficacy in this setting. AL (lumefantrine specifically) has also been shown to select for the *pfcrt* 76K wildtype [33]. However, the study found a significant increase of the mutant *pfcrt* CVIET haplotype over the study period. A similar trend has previously been observed in the same study area and QN usage was speculated to be the cause [29, 34]. QN is typically used at a suboptimal dosage (10 mg/kg × 2 for 3 days) in Bissau, and its effectiveness is < 50%. However, it may also be that the two observed events (2010–2012 and 2014–2016) of increasing levels of the CVIET haplotype represent “highs” in a more long-term fluctuation of this haplotype.

The levels of the *pfdhfr* IRN triple mutant found in this study (fluctuating between 73 and 92%) indicate selection of this haplotype since earlier studies were conducted (in 2004; prevalence of 41%) [35]. Likewise, the current levels of the *pfdhfr/pfdhps* quadruple mutant (33–51%) indicate selection since previous studies were conducted (15% quadruple mutant in 2004) [35]. Large scale use of IPTp may have contributed to this selection, since IPTp is the only SP-based treatment that is still recommended and implemented in Guinea-Bissau [19], apart from a very recent deployment of seasonal malaria chemoprevention (SMC by use of SP + amodiaquine) in a northern region of the country [36]. SP was never first-line treatment in Guinea-Bissau, but was recommended as second-line treatment from 1996 to 2007. Selection may also be caused by use of SP for self-treatment of malaria, the use of sulfamethoxazole–trimethoprim for bacterial infections, and finally it is also possible that quadruple mutants are imported from neighbouring countries where SP has been used as first-line treatment and where mutant haplotypes have historically been more prevalent than in Guinea-Bissau [37–39].

Importantly, the current study revealed no SNPs of concern in *pfk13* [40]. Combined with the previously published data regarding *pfk13* polymorphisms from the area [15], there are no signs of artemisinin selective pressure of the kind seen in South-East Asia and Suriname [3].

### False positivity and false negativity of RDTs—epidemiological insights

Approximately 74% of the positive RDTs were found PCR positive for the multicopy *Plasmodium* 18S subunit, indicating a diagnostic false positivity percentage of 26%. Patients may very well have had circulating antigens, and hence the test was not false to be positive, but with no circulating parasites the patient did not actually have malaria, in contrary to the diagnosis. There is of course a limit of detection that applies to the 18S PCR, as well as the possibility of DNA degradation over time. However, with the typical detection limit of RDTs of 100–200 parasites/µl, a false positivity rate of 26% is not surprising. Furthermore, the current study found only 1% of the tested negative RDTs to be PCR positive, and hence false negative. The limit of detection of the 18S PCR, as well as the possibility of DNA degradation also apply to this finding. The low percentage of false negative RDTs, however, is not surprising given the setting of the study: included patients all referred to the clinic with symptoms and children were included in the study. Previous studies on the current epidemiology of malaria in the area have found that there is a decrease in acquired immunity, and that even older adolescents react with symptoms. Lastly, recent surveys have shown a decreasing prevalence, and a high percentage of sub-patent infections were, therefore, not expected.

### Demographic trends

The demographic data obtained from routinely collecting used RDTs, indicate that there was less malaria during the 2014 transmission period, than during 2015 and 2016 transmission periods. According to rainfall data obtained from Bandim for the three seasons, an increase in rainfall from 2014 to 2015 was observed, which may have contributed to a rise in malaria cases (yearly rainfall was 941.2 mm in 2014, 1393 mm in 2015 and 983.9 mm in 2016) [41]. A country-wide long-lasting insecticide-treated bed net distribution campaign and subsequent follow-up study carried out in June and December 2014, confirmed the low prevalence in 2014 (1.3% amongst children aged 0–59 months and 0.7% amongst children 5–14 years) [42]. Furthermore, according to the inclusion data from our study, adults (patients ≥ 15 years) represent the majority of infections during transmission periods, with a significantly larger proportion of adults amongst RDT positive patients, than amongst the entire RDT-tested population and the general population of the country [26]. These findings correspond well with the trend of increasing median age of malaria patients previously described for the study area [17, 18].

## Conclusion

This study provides proof of concept for the use of RDTs for molecular surveillance of anti-malarial resistance through massively parallel amplicon sequencing with Illumina technology, while highlighting major challenges when doing so. Furthermore, the study provides evidence that there is a high frequency of the *pfmdr1* c. 86N, that the *pfcrt* CVIET haplotype has increased significantly over the course of the study, that the *pfdhfr/pfdhps* quadruple mutant has increased substantially in frequency since 2004, and that there are no accumulating SNPs in *pfK13* as of May 2017 in Bissau, Guinea-Bissau. Lastly, the study provides evidence as to how routine sampling of used RDTs combined with minimal patient data, can provide insight regarding basic demographic trends amongst the malaria patients.

## Additional file

**Additional file 1.** Additional tables.

## Data Availability

The datasets generated and analysed in the study are not publicly available, due to the ethical approval agreement, but sequencing data can be requested from one of the corresponding authors. The patient database will not be available.
