# Supplementary material for: Proof of concept: used malaria rapid diagnostic tests applied for parallel sequencing for surveillance of molecular markers of anti-malarial resistance in Bissau, Guinea-Bissau during 2014–2017
Source: Malar J. 2019 Jul 26;18:252. doi: 10.1186/s12936-019-2894-8 (PMC6660714; doi:10.1186/s12936-019-2894-8)
Supplement: Supplementary file 1 — Additional file 1. Additional tables. [file 12936_2019_2894_MOESM1_ESM.docx]

Additional file 1: Table S1 Primers and fragments amplified

| gene | fragment (genomic) | fragment (codons) | outer fw primer | outer reverse primer | nested fw primer | nested reverse primer |
| --- | --- | --- | --- | --- | --- | --- |
| *pfcrt* | 270-772 | 31-138 | crt.48.70.OF | crt.805.826.OR | crt.270.296.FiO | crt.750.772.RiO |
|  |  |  | TGACGAGCGTTATAGAGAATTAG | GATTGGATATTTCCAGTAGTTC | **TCGTCGGCAGCGTCAGATGTGTATAAGAGACAG**TGGCTCACGTTTAGGTGGAGGTTCTTG | **GTCTCGTGGGCTCGGAGATGTGTATAAGAGACAG**CAGGCATCTAACATGGATATAGC |
|  | | | | | | |
| *pfmdr1* | fragment 1 - 179-711 | 60-237 | mdr1.1.25.OF | mdr1.942.961.OR | mdr1.179.208.FiO | mdr1.682.711.RiO |
|  |  |  | ATGGGTAAAGAGCAGAAAGAGAA | ATGGATATAACTGAGGCACCATTA | **TCGTCGGCAGCGTCAGATGTGTATAAGAGACAG**CATTTGTATGTGCTGTATTATCAGGAGGAA | **GTCTCGTGGGCTCGGAGATGTGTATAAGAGACAG**AGCCTCTTCTATAATGGACATGGTATTGTT |
|  |  | | | | | |
|  | fragment 2 - 3067-3780 | 1022-1260 | mdr1.2908.2935.OF | mdr1.3892.3921.OR | mdr1.3067.3094.FiO | mdr1.3754.3780.RiO |
|  |  |  | TTTGCATTTAGTTCAGATGATGAAATG | TGGTCCAACATTTGTATCATATTTATTTGG | **TCGTCGGCAGCGTCAGATGTGTATAAGAGACAG**AGAATTATTGTAAATGCAGCTTTATGGG | **GTCTCGTGGGCTCGGAGATGTGTATAAGAGACAG**TAACATGGGTTCTTGACTAACTATTGA |
|  | | | | | | |
| *pfdhfr* | 35-523 | 12-174 | dhfr.1.29.OF | dhfr.630.659.OR | dhfr.35.62.FiO | dhfr.493.523.RiO |
|  |  |  | ATGATGGAACAAGTCTGCGACGTTTTCGA | GTTGTATTGTTACTAGTATATACATCGCTA | **TCGTCGGCAGCGTCAGATGTGTATAAGAGACAG**ATGCCATATGTGCATGTTGTAAGGTTGA | **GTCTCGTGGGCTCGGAGATGTGTATAAGAGACAG**CTAAAAATTCTTGATAAACAACGGAACCTCC |
|  | | | | | | |
| *pfdhps* | 1177-1868 | 392-622 | dhps.1061.1089.OF | dhps.1916.1944.OR | dhps.1177.1203.FiO | dhps.1843.1868.RiO |
|  |  |  | ACAAATATGTGAGTAGGATGAAAGAACAA | CATCCAATTGTGTGATTTGTCCACAATAT | **TCGTCGGCAGCGTCAGATGTGTATAAGAGACAG**GGAATATTAAATGTTAATTATGATTCT | **GTCTCGTGGGCTCGGAGATGTGTATAAGAGACAG**ATTACAACATTTTGATCATTCATGCA |

Additional file 1: Table S1 continued

| *pfK13* | fragment 1 - 50-498 | 17-166 | K13.1.29.OF | K13.1023.1047.O.R | K13.50.76.FiO | K13.471.498.FiO |
| --- | --- | --- | --- | --- | --- | --- |
|  |  |  | ATGGAAGGAGAAAAAGTAAAAACAAAAGC | TCTTCATCAAATCGTTTCCTATGTT | **TCGTCGGCAGCGTCAGATGTGTATAAGAGACAG**CTATGACGTATGATAGGGAATCTGGTG | **GTCTCGTGGGCTCGGAGATGTGTATAAGAGACAG**GTTGGTATTCATAATTGATGGAGAATTC |
|  |  | | | | | |
|  | fragment 2 - 427-914 | 143-304 |  |  | K13.427.455.FiO | K13.890.914.RiO |
|  |  |  | same as *pfk13* fragment 1 | same as *pfk13* fragment 1 | **TCGTCGGCAGCGTCAGATGTGTATAAGAGACAG**CTGACAGCAAATAATATAACTAATAATCT | **GTCTCGTGGGCTCGGAGATGTGTATAAGAGACAG**TCTTCATCAAATCGTTTCCTATGTT |
|  |  | | | | | |
|  | fragment 3 - 803-1207 | 267-402 | K13.764.794.O.F | K13.2145.2167.OR | K13.803.831.FiO | K13.1178.1207.RiO |
|  |  |  | GAGTACGATTGTACAAAGAATTAGAAAACCG | GCTATTAAAACGGAGTGACCAAATCTG | **TCGTCGGCAGCGTCAGATGTGTATAAGAGACAG**TTGAAGAACAGAAATTACATGATGAAAGA | **GTCTCGTGGGCTCGGAGATGTGTATAAGAGACAG**ATAACTCACTATCCCTATCTAAGAATATTC |
|  |  | | | | | |
|  | fragment 4 - 1139-1669 | 380-556 |  |  | K13.1139.1166.FiO | K13.1642.1669.RiO |
|  |  |  | same as *pfk13* fragment 3 | same as *pfk13* fragment 3 | **TCGTCGGCAGCGTCAGATGTGTATAAGAGACAG**TAAGTGGAAGACATCATGTAACCAGAGA | **GTCTCGTGGGCTCGGAGATGTGTATAAGAGACAG**CTTCTACATTCGGTATAATAGAAGAGCC |
|  |  | | | | | |
|  | fragment 5 - 1637-2127 | 546-709 |  |  | K13.1637.1663.FiO | K13.2100.2127.RiO |
|  |  |  | same as *pfk13* fragment 3 | same as *pfk13* fragment 3 | **TCGTCGGCAGCGTCAGATGTGTATAAGAGACAG**ATGATGGCTCTTCTATTATACCGAATG | **GTCTCGTGGGCTCGGAGATGTGTATAAGAGACAG**CCAAGCTGCCATTCATTTGTATCTGGT |

Additional file 2: Table S2 Control sample data

|  | *pfcrt* | *pfmdr1* | *pfdhfr* | *pfdhps* |
| --- | --- | --- | --- | --- |
|  | c. 72-76 | c. 86, 184, 1034, 1042, 1246 | c. 51, 59, 108, 164 | c. 436, 437, 540, 581, 613 |
| AA | CVMNK | NYSND | NRNI | AAKAA |
| 164L | CVIET | YYSND | IRNL | SGKAT |
| 540E | ----- | NYSND | IRNI | AAKAA |
| AG | CVMNK | N/YFSND | NRNI | AGKGS |
| mra1239 | CVIET | NYSND | IRNL | SGEAS |
| mra1238 | CVIET | NYSND | IRNI | AGEAA |
| 7g8 | SVMNT | NFCDY | ICNI | SGKAA |
| k1 | CVIET | YYSND | NRNI | SGKGA |
| dd2 | CVIET | YYSND | IRNI | SGKAS |
| fcr3 | CVINT | YYSND | NCTI | SAKAA |
| 3d7 | CVMNK | NYSND | NCSI | SGKAA |
